# Arduino-based fine particulate matter STEM program: enhancing problem-solving and collaboration in a post-pandemic blended high school setting

**DOI:** 10.3389/fpsyg.2025.1524777

**Published:** 2025-04-22

**Authors:** Sejun Oh

**Affiliations:** Department of Mathematics Education, Hongik University, Seoul, Republic of Korea

**Keywords:** Arduino-based STEM, blended learning, fine particulate matter pollution, problem-solving skills, collaborative learning

## Abstract

After the pandemic, the need for research on teaching and learning methods that promote motivation, engagement, and soft skills in blended learning environments has increased. This study presents an Arduino-based STEM program centered on fine particulate matter measurement to enhance high school students’ problem-solving abilities and collaborative thinking. Conducted in a blended learning setting, the program guided learners to build fine particulate matter sensors, collect real air quality data, and discuss potential solutions at personal, community, and national levels. The research utilized a mixed-methods approach, analyzing quantitative pre-and post-surveys (48 items) and qualitative interviews and reflection reports. Quantitative analysis results showed significant improvements in math/science self-efficacy (e.g., “I quickly understand scientific concepts,” *p* = 0.001, Cohen’s *d* = 0.80) and collaboration (e.g., “I actively exchange opinions in math classes,” *p* < 0.05). Qualitative analysis revealed that students felt more confident debugging block-based codes and recognized the social relevance of scientific data. Additionally, many students expressed increased awareness of fine particulate matter and environmental issues and a willingness to address real-life problems using computing tools. These findings highlight the importance of integrating real-world STEM programs into blended learning environments post-pandemic. By harmonizing technical skills (Arduino assembly, data analysis) with soft skill development (communication, teamwork), the program inspired learners to perceive STEM as both practical and interdisciplinary.

## 1 Introduction

The outbreak of COVID-19 has triggered a significant transformation in the educational landscape, particularly with the widespread adoption of blended learning, which combines face-to-face and remote education. Recent studies suggest a significant rise in blended learning implementation across various countries following the pandemic. For instance, the National Education Association reported that 72% of U.S. public schools integrated blended learning strategies in 2023, while Japan’s Ministry of Education, Culture, Sports, Science, and Technology (MEXT) indicated that 82% of Japanese high schools plan to adopt blended learning by 2024 (Ministry of Education, Culture, Sports, Science, and Technology, 2024). Similar trends have been observed in other regions, reflecting a broader move toward flexible and technology-enhanced educational models. This shift has necessitated teachers and students adapting to new learning environments and approaches (Bates, 2017).

STEM education emphasizes the integration of multiple disciplines, enabling students to solve real-world problems through scientific inquiry and creative critical thinking (Borba et al., 2016). This approach moves beyond traditional knowledge transfer, guiding students to explore, collect data, analyze findings, and present solutions in a process that nurtures scientific reasoning and creativity. Environmental issues, mainly complex and multifaceted, like air pollution, are areas where STEM education’s interdisciplinary nature is particularly valuable.

In this context, STEM education has shown promise in developing students’ self-directed learning and integrated thinking skills (Graham, 2006). This is particularly relevant for South Korean high schools, where the national curriculum strongly emphasizes developing scientific literacy and problem-solving skills. However, the PISA 2022 results on students’ creative thinking reveal significant disparities across ability groups. In the scientific problem-solving domain, high-level students showed an average correctness rate of 71.68%, compared to only 33.11% among low-level students. A similar gap was noted in social problem-solving, where the high-level group achieved a correctness rate of 78.11%, whereas the low-level group lagged behind at 24.29% (Kim et al., 2024).

In school environments where students spend a significant portion of their day, the effects of fine particulate matter are increasingly evident and directly impact learning conditions. Fine particulate matter pollution has become an increasingly urgent environmental concern in South Korea, as illustrated by recent analyses of PM2.5 concentrations from January 2015 to December 2020 at three national background monitoring sites—Baekryeongdo, Ulleungdo, and Jeju (Im et al., 2021). Despite the remote nature of these locations, recent measurements indicate that PM2.5 concentrations surpass both the revised 2018 National Ambient Air Quality Standard (15 μg/m^3^) and the WHO guideline (10 μg/m^3^). Prolonged exposure to elevated PM2.5 levels, particularly during school hours, has been linked to decreased cognitive function and increased health risks (Ghosh et al., 2024; Lu et al., 2021). Consequently, fine particulate matter emerges as a significant factor influencing students’ academic performance, health, and overall quality of life. It is therefore critical that learners understand the seriousness of fine particulate matter pollution and develop the readiness to address it proactively.

One approach that can foster such engagement is that an educational program be developed whereby students construct and operate their own fine particulate matter measurement devices using Arduino, thereby enabling them to collect real-time data and analyze local conditions. Previous investigations that have examined Arduino-based STEM education (Karaahmetoğlu and Korkmaz, 2019; Kim et al., 2020) have primarily focused on coding proficiency or on enhancing general broad problem-solving skills. Although research that explores the use of Arduino for tackling environmental issues (Lezcano Sirre, 2024), has been conducted, studies that concentrate on problem‐solving and collaboration aspects remain scarce. Specifically, there is a need for research that investigates how Arduino can be employed to address fine particulate matter issues in high schools’ blended learning environments. This study is designed to develop and implement a STEM program that utilizes Arduino. The research questions are as follows:

(1) What is the impact of a STEM program that incorporates Arduino in a blended learning environment on high school students’ motivation and interest in learning?
(2) To what extent does a STEM program that integrates Arduino within a blended learning environment enhance students’ capabilities in solving real‐world environmental problems?
(3) How does a STEM program that employs Arduino in a blended learning setting affect the way students communicate within teams?

By exploring these questions, this research seeks to offer insights that elucidate the effectiveness of STEM education in a blended learning environment. Moreover, it provides practical strategies that educators can employ to enhance student engagement and interdisciplinary learning, particularly when tackling real‐world issues such as environmental pollution. The findings of this study are anticipated to add to the expanding body of research on STEM education and to guide future curriculum development in an educational context that has evolved following the pandemic. This study was carried out over one semester and involved a single class of 20 high school students in a blended learning environment that utilized MS TEAMS as the LMS platform, with a focus on activities that measure and analyze fine particulate matter. Therefore, although the results of this study might not be generalized to every educational setting, they can provide valuable insights for implementing similar programs in comparable environments.

## 2 Theoretical background

### 2.1 Soft skills in education

Soft skills such as problem-solving, collaboration, and critical thinking are essential (Griffin and Care, 2015; National Research Council, 2012). These skills enable individuals to solve complex problems in rapidly changing environments collaboratively. Problem-solving considers whether individuals analyze given conditions and information, explore appropriate solution strategies, reflect on the process to derive valid results according to procedures, engage actively and confidently in problem-solving, and persistently challenge themselves to find appropriate methods. Collaboration includes coordinating tasks within a team, effectively managing conflicts, providing constructive feedback, and building trust among team members to make consensus-based decisions for achieving shared goals. Critical thinking involves analyzing given information, recognizing patterns, and making evidence-based rational judgments (Trilling and Fadel, 2009).

Recently, “self-regulation” and “communication” have been emphasized as important soft skills (Bernard et al., 2014). Self-regulation refers to managing time and adjusting behavior according to progress. At the same time, communication considers whether individuals understand others’ views, recognize the convenience of expressions, and respect and consider others’ opinions. These soft skills have been shown to enhance student engagement and knowledge acquisition (National Research Council, 2012). This study aims to investigate the correlation between soft skills and an Arduino-based STEM program addressing fine particulate matter in a blended learning environment.

### 2.2 Blended learning: concepts and characteristics

Blended learning integrates face-to-face and online education, offering advantages in overcoming the constraints of time and space (Hrastinski, 2019). It combines synchronous (real-time) and asynchronous (self-paced) elements, providing flexibility and adaptability to diverse learning needs. Well-designed blended learning environments can significantly improve learning outcomes compared to traditional face-to-face education (Bernard et al., 2014; Means et al., 2013).

Several features of blended learning have been identified as beneficial for students. For instance, digital tools can provide interactive platforms and timely feedback, course designs can balance online and offline tasks, and active learning strategies may include collaborative or problem-based activities (Bernard et al., 2014; Graham, 2006).

By emphasizing learner-centered approaches, blended learning can offer additional learning time, more varied resources, and opportunities for peer interaction, which some researchers associate with higher motivation and better content understanding (Garrison and Vaughan, 2008; Means et al., 2013). Nevertheless, neither source explicitly addresses “autonomy” and “scaffolding” as core components of blended learning, suggesting the need for clearer explanations of how these concepts fit within a blended framework.

Additionally, while some meta-analyses do explore how blended environments can foster self-regulated learning (Bernard et al., 2014; Kintu et al., 2017), the direct link to broader soft skills—such as communication or collaboration—is less frequently detailed. In studies where learners must manage their time and interact in diverse modalities, self-regulation is often highlighted as a critical factor for success. Blending online and face-to-face methods can align well with many of the soft-skill competencies (e.g., self-regulation) discussed in Section 2.1, but additional evidence is required to substantiate blended learning’s broader impact on autonomy, scaffolding, and the full spectrum of soft skills.

### 2.3 STEM education: principles and impact

As discussed in Section 2.1, soft skills such as problem-solving, collaboration, and critical thinking are crucial for today’s learners. These skills play an especially vital role in STEM education, intersecting seamlessly with the technical and content-based competencies of science, technology, engineering, and mathematics (Bybee, 2013; English, 2016; Kelley and Knowles, 2016). Whereas blended learning (see Section 2.2) provides a flexible instructional approach, STEM education furnishes an integrated curriculum that addresses real-world challenges and interdisciplinary thinking, fostering content mastery and soft skill development.

#### 2.3.1 Interdisciplinary focus and authentic contexts

STEM education unifies the traditionally siloed subjects of science, technology, engineering, and mathematics into authentic learning tasks. Through problem-based and project-based activities, students can enhance their collaboration, creativity, and logical reasoning skills while learning how to apply academic knowledge to real-world problems (Moore et al., 2014; Tseng et al., 2013). By tackling real-world issues such as designing environmental monitoring devices or constructing sustainable structures, students experience the application of classroom knowledge in practical contexts. These activities increase student engagement and promote deeper learning (Bybee, 2013).

#### 2.3.2 Development of soft skills in STEM

One advantage that STEM education offers is its potential to nurture the development of soft skills. For instance, in STEM education, students are often involved in processes that include formulating hypotheses, analyzing data, and that entail finding solutions to complex problems. Students investigate multiple possibilities, thereby enhancing their critical thinking abilities and cultivating a resilient mindset that is essential for addressing challenges (English, 2016). Moreover, team‐based projects that promote effective communication and collaborative decision‐making are essential for the successful completion of STEM projects (Kelley and Knowles, 2016). STEM environments also offer learners opportunities that allow them to identify variables from real‐world data, to synthesize knowledge from multiple disciplines, and to engage in critical reflection. This process that enables them to grasp the essence of problems and to critically assess potential solutions is beneficial (Tseng et al., 2013).

#### 2.3.3 Empirical evidence of STEM effectiveness

STEM‐based education programs that are implemented in academic settings present several advantages. Among these advantages, this work explores the connections that exist between STEM education and enhanced academic achievement and engagement, improved problem‐solving skills, and greater career readiness.

##### 2.3.3.1 Enhanced achievement and engagement

STEM programs can inspire interest and motivation for learning, potentially leading to improved performance in science and mathematics. A meta-analysis by Becker and Park (2011) revealed that STEM approaches generally positively impact student learning outcomes, confirming the effectiveness of integrating STEM into educational practices. While Tseng et al. (2013) emphasized positive attitudes toward STEM in a project-based learning context, direct correlations with academic achievement were less explicit. By contrast, Becker and Park (2011) provide a broader quantitative overview, suggesting that increased engagement in STEM activities can relate to measurable gains in content mastery.

##### 2.3.3.2 Stronger problem-solving abilities

Integrated STEM curricula aim to cultivate higher-order thinking skills by involving students in real-world, interdisciplinary challenges (Moore et al., 2014; Stohlmann et al., 2012). Stohlmann et al. (2012) specifically discuss how engineering-infused science instruction can prompt learners to define, analyze, and address complex problems more effectively, though robust, quantitative data on the magnitude of problem-solving gains remains limited. While English (2016) underscores the need for integrated STEM, empirical evidence directly connecting STEM interventions to problem-solving improvements is still evolving. Nevertheless, studies such as Stohlmann et al. (2012) and Becker and Park (2011) collectively hint at a trend: as students engage in design-based or interdisciplinary STEM projects, their ability to structure and test solutions to authentic problems appears to strengthen.

##### 2.3.3.3 Career readiness

Hands-on, collaborative STEM experiences may increase student awareness of STEM-related careers, potentially boosting their confidence to pursue post-secondary STEM programs or enter the workforce with stronger teamwork and technical skills (Kelley and Knowles, 2016). However, direct empirical links between K–12 STEM activities and long-term career success (e.g., job adaptability, innovation skills) are less systematically documented. For example, Kelley and Knowles (2016) argue that integrated STEM builds a foundation for 21st-century workforce demands but do not offer large-scale longitudinal data to confirm how these skills translate to on-the-job performance. In response, some policy-oriented publications, like the National Research Council (2011) report, suggest that sustained exposure to integrative STEM experiences can nurture a STEM-capable workforce, but more cohesive, longitudinal studies remain necessary.

#### 2.3.4 Synergy with blended learning

STEM education can be enriched further by blended learning strategies (see Section 2.2), as digital tools and virtual collaborations enable extended inquiry, asynchronous group work, and customized feedback loops. This dual emphasis on hands-on, interdisciplinary tasks and flexible instructional modalities highlights the complementary relationship between STEM education and blended learning approaches.

### 2.4 Environmental importance of fine particulate matter pollution

Fine particulate matter pollution, which is often quantified as PM2.5, is known to pose significant health and cognitive risks. Studies indicate that if PM2.5 levels surpass recommended thresholds, students may encounter diminished cognitive function, an increased risk of respiratory diseases, and higher rates of absenteeism (Im et al., 2021). Fine particulate matter pollution is a critical issue that is closely related to students’ everyday lives, thereby underscoring the need for educational initiatives on this subject. Topics concerning fine particulate matter pollution offer opportunities that encompass monitoring, data interpretation, and scientific inquiry. Several studies suggest that monitoring programs that are implemented in educational settings can raise students’ awareness of environmental issues and deepen their scientific understanding (Bhang and Huh, 2023; Kim et al., 2020). For instance, when students measure PM2.5 levels on campus and analyze real‐time data, they are enabled to develop data utilization skills. Additionally, by investigating local air quality issues and proposing practical, actionable solutions, students are able to cultivate their critical thinking and collaborative problem‐solving abilities. Moreover, participating in data collection activities that are related to real environmental problems has been demonstrated to motivate students to explore the causes of fine particulate matter and to investigate methods for mitigating its effects. For example, Kim et al. (2020) provided evidence that an elementary‐level fine particulate matter education program substantially increased students’ interest in and commitment to preventive measures. Similarly, an international comparative study by Bhang and Huh’s (2023) found that environmental education that focuses on fine particulate matter effectively enhances students’ environmental awareness, irrespective of regional differences.

### 2.5 Arduino-based environmental monitoring: educational potential

Arduino is a tool that can be effectively employed in blended learning environments, which serves to strengthen soft skills and to address real‐world issues such as fine particulate matter pollution through STEM‐based approaches. By assembling, programming, and testing Arduino devices that are equipped with PM2.5 sensors, students can obtain valuable experiences that serve to enhance their critical thinking, teamwork, and problem‐solving skills.

#### 2.5.1 Overview of Arduino in education

Arduino has garnered attention that spans primary, secondary, and higher education, owing to its ease of use in prototyping solutions for real‐world problems. The accessibility of the platform that permits students to create practical projects bridging theoretical knowledge and hands‐on application is noteworthy. For example, the FLEx (Forward Learning Experience) program that has engaged students in interactive circuit building serves to illustrate Arduino’s utility in large‐scale outreach initiatives (Evans and Schares, 2017). Studies indicate that incorporating Arduino Kits in project‐based classrooms that are designed to foster engagement can boost student interest and enhance teamwork (Hambali et al., 2024). Furthermore, research involving visual programming languages (e.g., Scratch) that integrates Arduino‐based robotics demonstrates that abstract coding concepts become more tangible (Santos et al., 2024). This hands‐on integration that combines theoretical learning with practical application can serve to bolster engagement, computational thinking, and technological literacy.

#### 2.5.2 Integrating soft skills and STEM

When learners utilize Arduino boards that are used to collect real‐time environmental data such as temperature, humidity, or PM2.5, they are required to collaborate on circuit design, troubleshoot mechanical or coding issues, and analyze the resulting outcomes. These activities that involve both collaborative and technical tasks simultaneously reinforce soft skills, such as communication and conflict resolution, while also strengthening technical proficiencies like sensor calibration and code debugging. By designing and iterating monitoring devices that allow them to integrate science, technology, engineering, and mathematics in a real‐world context, students thereby deepen their engagement with STEM (Herro et al., 2023).

#### 2.5.3 Blended learning advantages

Arduino projects that combine online tutorials, open‐source libraries, and in‐person lab tasks naturally align with a blended learning model. According to researchers, this approach that integrates both virtual and physical learning environments not only provides students with real‐world hardware experience but also enriches STEM education (Papadimitropoulos et al., 2021). Students can explore circuit diagrams asynchronously that enable self‐directed learning and then come together to assemble and test devices during in‐person collaborative sessions. The integration of asynchronous learning with hands‐on experiments in a blended learning environment that optimizes resources such as time, space, and cost has proven effective for skill development (Nayak et al., 2022). This approach that allows students to practice at home or in the classroom facilitates their experience of the core elements of blended learning, which include self‐directed learning processes, immediate peer feedback, and iterative improvement. Moreover, it has been reported that low‐cost Arduino‐based tools can lead to improvements of up to 1.5% over traditional learning experiences, thereby emphasizing their practicality in enhancing conceptual understanding (Devarajan et al., 2023).

#### 2.5.4 Addressing fine particulate matter pollution

Utilizing Arduino that allows learners to easily create devices for measuring PM2.5 levels serves to make environmental science more accessible to K-12 students. For instance, researchers have designed portable outdoor air quality measurement systems that employ Arduino to detect particulate matter (Gunawan et al., 2018). By extending these ideas into school settings, students that are involved in such projects transform abstract pollution concepts into tangible, local data, which they use to discuss trends, propose solutions, and advocate for improved air quality. Such hands‐on sensor integration that merges practical activity with data analysis encourages a deeper insight into both the technical and social aspects of environmental issues.

#### 2.5.5 Practical implications and future directions

Empirical evidence from school‐based programs that have incorporated Arduino‐based activities indicates that such activities spark student interest in engineering and design while bolstering problem‐solving skills. Research on project‐based physics learning that utilizes Arduino indicates that there are improvements in collaboration and real‐world problem‐solving outcomes (Dwinda et al., 2024). Future studies that investigate how fine particulate matter monitoring with Arduino influences students’ long‐term attitudes toward STEM careers and community engagement may provide further insights. By integrating soft skills, blended learning, rigorous STEM content, and pressing air quality concerns, Arduino‐centered programs are capable of profoundly influencing the manner in which students learn and apply scientific knowledge.

## 3 Methodology

The study aimed to develop and implement a STEM program focusing on “Measuring Fine particulate matter Using Arduino and Exploring Solutions” in high school settings. Through approximately 1 year of preparatory work, collaborative design by expert teachers, and a mixed-methods evaluation, this study examined how the program enhanced students’ learning motivation, problem-solving abilities, and collaborative thinking skills.

### 3.1 Program development

The program was developed through weekly collaborative meetings of five STEM teachers specializing in Mathematics, Biology, Earth Science, Social Studies, and Art. These teachers formed a dedicated research group to ensure subject-specific accuracy and integrate interdisciplinary perspectives. Regular meetings ensured coherence among the subjects, with each teacher contributing:

- Biology for health impacts of fine particulate matter
- Earth Science for its physical properties
- Social Studies for environmental policies
- Mathematics for data analysis
- Art for visualization techniques

In addition to referencing integrated STEM education models (e.g., National Research Council, 2012) and design-based learning principles (Choi et al., 2012; Kim et al., 2015), teachers co-taught each session and provided iterative feedback. For instance, they identified time constraints and difficulty levels after the first two sessions, prompting minor adjustments to the pacing and materials. This dynamic approach ensured that research-based practices informed the content and pedagogy throughout.

#### 3.1.1 Session 1: constructing a fine particulate matter measuring device using Arduino

- Students began by learning about fine particulate matter definitions and its generation mechanisms. They then used Arduino microcontrollers and fine particulate matter sensors to build a measuring device. Emphasis was placed on designing algorithms in a block-based environment (mBlock), enhancing computational thinking and problem-solving skills.

#### 3.1.2 Session 2: measuring fine particulate matter concentrations and data collection

- Using the device built in Session 1, students collected in-classroom dust concentration data. They manipulated variables (e.g., ventilation), observing environmental changes. This laid a practical foundation for analyzing real environmental issues.

#### 3.1.3 Session 3: discussing solutions to reduce fine particulate matter

- In this session, students used MS Teams to share the measurement data collected in the previous sessions, collaboratively analyze the findings, and propose creative solutions for reducing fine particulate matter at personal, regional, and national levels. By working within a shared online document (e.g., a collaborative file or channel in MS Teams), each group member contributed their ideas and data interpretations in real time, regardless of their physical location. This setup not only promoted analytical thinking and collaboration but also reinforced soft skills linked to problem-solving and communication.

#### 3.1.4 Session 4: debate on national standards for fine particulate matter

- The final session featured a debate on the topic, “Should South Korea Strengthen Its Fine Particulate Matter Standards?” versus “Should the Current Standards Be Maintained?” Students drew on the MS Teams-based measurement data repository and collaborative solution outlines from Session 3 to support their arguments. Using screen-sharing and real-time updates in MS Teams, they effectively presented evidence, challenged opposing views, and refined their points throughout the debate. This environment fostered critical thinking, communication skills, and scientific inquiry in a blended learning context. Throughout, the emphasis remained on problem-solving over merely technical coding mastery, allowing students to focus on addressing real-world issues in a collaborative, partially or fully remote setting.

### 3.2 Participant selection

This program targeted one first-year high school class (20 students), chosen based on classroom capacity, limited Arduino kits, and the absence of an information technology course at the school. All students had no prior Arduino experience, confirmed through a preliminary questionnaire. While initially planned for multiple classes, the single-class approach enabled in-depth observation and iterative feedback. However, this design lacks a formal comparison (control) group, limiting generalizability of the results.

### 3.3 Data collection

Given the study’s focus on program effectiveness, we employed a mixed-methods approach (Creswell and Plano Clark, 2017) to capture both measurable changes in student performance (quantitative) and nuanced aspects of engagement, perceptions, and soft-skill development (qualitative).

#### 3.3.1 Quantitative data collection

We conducted pre-post surveys featuring:

- Program Impact Survey (PP1_x ~ PP2_x): 40 items (4-point Likert), measuring scientific understanding, technology interest, and collaboration skills.

Example: “I find the content of science class interesting.”

Some items (e.g., PP2_27, PP2_35) indirectly assess problem-solving self-efficacy, while others (e.g., PP1_12, PP2_22) relate to collaboration or communication.

- Subject Preference & Achievement (PP3_x, PP4_x): 8 items (5-point scale), capturing interest and perceived achievement in core subjects (Math, Science).
- Program Satisfaction (Q1 ~ Q15): 15 items (5-point scale), measuring overall STEM experience satisfaction.

Paired-sample t-tests compared pre- and post-scores; *p*-values and Cohen’s d indicated effect size. Statistical analysis revealed significant improvements (*p* < 0.05) in several areas, especially math problem-solving (Cohen’s *d* = 0.637) and science content (Cohen’s *d* = 0.804).

#### 3.3.2 Qualitative data collection

To examine student perceptions in depth, we gathered:

A semi-structured interview protocol was used with each participant. Although the conversation allowed for follow-up and probing questions, three core questions were asked to all participants in a structured manner:

OE1: “What is the biggest difference between this STEM class and your previous classes?”

OE2: “Which part of the STEM activities did you find most challenging?”

OE3: “What aspects of the class did you find most beneficial or enjoyable?”

Responses to these three standardized questions were then coded collectively as “open-ended items” (OE1, OE2, OE3). Coded categories (e.g., “student-centered tasks,” “collaborative learning,” “problem-solving approach”) were derived inductively from the data and verified through cross-checks among the author and collaborating teachers involved in program development. Frequencies and mean ranks of participant responses now appear in Tables 1–3 of the Results section, illustrating how students perceived differences (OE1), challenges (OE2), and positive aspects (OE3). Although the class consisted of 20 students, some participants did not respond to certain questions, while others provided multiple responses. Percentages reported in Tables 1–3 were calculated based on the total number of students (N = 20), regardless of missing or multiple responses.

**Table 1:** OE1 “Differences from Previous Classes.”

Category/response	Frequency	Percentage	Rank
OE1_1 Many group tasks	10	50%	#1 difference
OE1_2 Student-centered approach, less lecture	2	10%	Tied #2
OE1_3 Linking multiple subjects (Math, Science, Tech, etc.)	2	10%	Tied #2
OE1_4 Other (e.g., environmental context, coding emphasis)	4	20%	

**Table 2:** OE2 “Challenges.”

Category/response	Frequency	Percentage	Rank
OE2_1 Time shortage for building & coding	11	55%	#1 challenge
OE2_2 Difficulties with Arduino wiring/debugging	7	35%	#2
OE2_3 Conflicting opinions in group tasks	2	10%	
OE2_4 Other (e.g., sensor accuracy concerns, advanced math needed)	1	5%	

**Table 3:** OE3 “Positive Aspects.”

Item (OE3_x)	1st	2nd	3rd	4th	5th	6th	Mean rank
OE3_1 Learning multiple subjects together, e.g., math, science, technology	4	3	4	3	4	2	3.3
OE3_2 Student-centered activities; minimal teacher lecture	5	5	0	7	1	2	3
OE3_3 Plenty of group activities, collaborating with classmates	6	5	5	0	3	1	2.6
OE3_4 Encourages self-directed thinking and learning	3	2	6	3	2	4	3.55
OE3_5 Can see how what we learn applies to real life	1	3	3	6	6	1	3.8
OE3_6 Opportunities to get information on science/tech careers	1	2	2	1	4	10	4.75

Reflection reports: After each session, students documented their design processes, challenges, and personal insights. A thematic analysis identified evidence of problem-solving steps (definition, strategy, implementation, reflection), collaboration behavior, and awareness of fine particulate matter.

Project artifacts: Construction logs and sensor data, providing tangible evidence of iterative problem-solving and practical technical skills.

Data on students’ soft skills was triangulated through survey data, interviews, reflections, and artifacts to enhance validity. The problem-solving rubric was developed during the implementation process to more rigorously capture iterative trials.

#### 3.3.3 Fine particulate matter data collection and analysis

Students used Arduino-based sensors to gather real-time fine particulate matter readings in their classroom. This hands-on procedure served two roles: (1) giving environmental monitoring exposure and (2) furnishing evidence-based debate topics. Teachers verified measurement accuracy and aligned it with subject-specific knowledge. Reflection reports confirmed that active sensor use deepened students’ grasp of technical measurement and environmental impact.

Moreover, teachers played a continuous co-teaching role, identifying logistical obstacles (time, material readiness) and adjusting the program’s pacing. After the 4th session, teacher-student feedback informed modifications to session guidelines and future curriculum expansions.

## 4 Results

This study assessed the impact of a STEM program focused on “Measuring Fine particulate matter Using Arduino and Exploring Solutions” on high school students’ learning motivation, problem-solving abilities, and collaborative thinking. In addition to quantitative pre-post surveys, we conducted qualitative coding of interviews, reflection reports, and open-ended (OE) responses to capture students’ perspectives on STEM activities, differences from previous classes, and challenges encountered.

### 4.1 Overview of the program implementation

The STEM program integrated Arduino-based fine particulate matter measurement with theoretical knowledge, aiming to increase student engagement and problem-solving while highlighting creative approaches over purely technical coding. Table 4 summarizes the program’s structure from initial discussions on fine particulate matter to final debates on national standards.

**Table 4:** STEM program overall table.

Period	Activities	Core emphasis	Tools & methods (blended learning)
Period 1	Use Arduino and a fine particulate matter sensor to create a measuring device (algorithmic thinking).	Introduce Arduino basics; emphasize coding logic, problem-solving, and sensor assembly.	In-person: Hands-on construction of sensors; brief digital resources for reference (e.g., tutorial videos).
Period 2	Measure dust concentrations in the classroom; manipulate variables (data analysis).	Collect real-time data; learn math/statistical concepts by calculating mean/SD and analyzing environmental changes.	In-person: Sensor usage in the classroom. Some sharing of initial results via MS Teams channels for data logging.
Period 3	Discuss solutions to reduce fine particulate matter (creative design).	Collaboration and analytical thinking: propose personal, regional, and national solutions.	MS Teams for real-time group discussions and collaborative document editing. Slide creation and brainstorming online.
Period 4	Debate on national dust standards, referencing real data (critical thinking).	Engage in structured debate on “Should South Korea Strengthen Its Fine Particulate Matter Standards?” using collected sensor data.	MS Teams for sharing final data in a collaborative file and real-time referencing during the debate. Mixed in-person debate.
Emotional experience	Measure dust levels to sense the seriousness of the problem; discuss multi-level solutions (individual, regional, national) to foster community awareness.	Reinforce real-world relevance, cultivate empathy and responsibility for environmental issues.	Combined in-person + MS Teams postings of personal reflections and ongoing discussions.

Students watched a short video on fine particulate matter, reviewed respiratory health in biology, and then constructed Arduino measuring devices (using “mBlock” block coding). Activities included:

Development Period 1 (50 min): Hands-on creation of the dust sensor kit, primarily conducted in person.

Development Period 2 (50 min): Examining factors that influence dust levels; calculating mean/SD using Excel or Grapher. Students began to share initial datasets through MS Teams for asynchronous review.

Development Period 3 (50 min): Using MS Teams, groups collaboratively discussed multi-scale dust solutions and created shared slides or documents. This approach allowed all students to access and edit the same files in real time, no matter their location, and to brainstorm creative design solutions for reducing fine particulate matter.

Development Period 4 (50 min): A debate on WHO vs. national fine particulate matter standards, incorporating MS Teams as a reference platform for updated sensor data and previously drafted solutions. Students practiced critical thinking and communication, seamlessly toggling between face-to-face debate and online collaborative notes.

**Figure 1:**
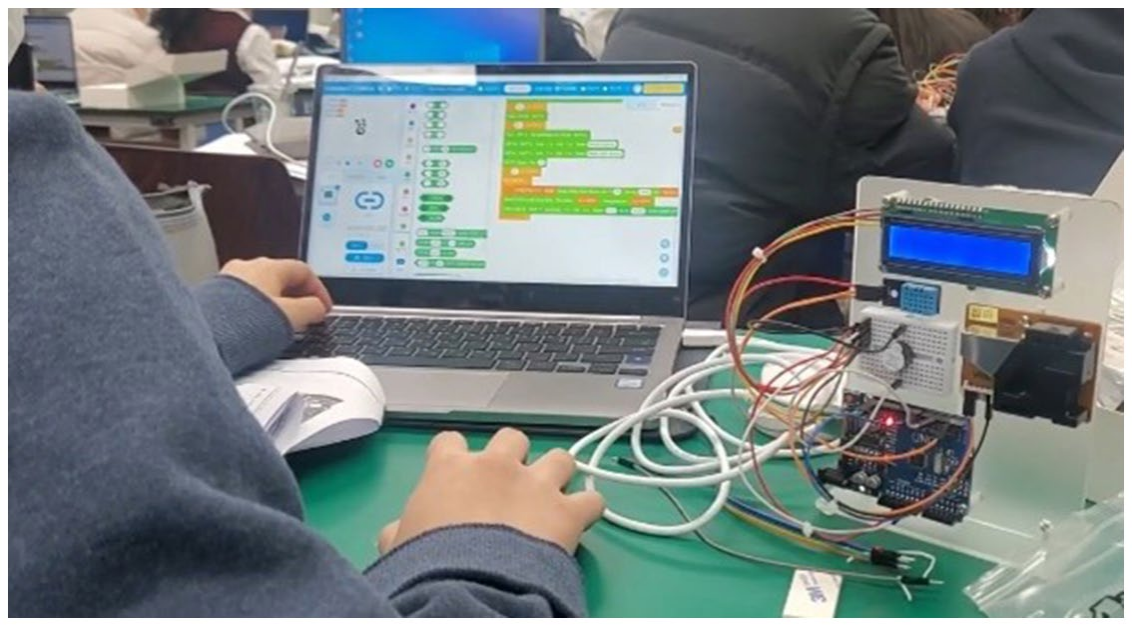
Programming the Arduino device using mBlock software.

In Figure 1, students actively code an Arduino microcontroller and observe real-time sensor outputs.

Upon finishing these main sessions, students summarized their experiences and reflected on fine particulate matter’s seriousness, noting both personal efforts and broader policy implications in a digital reflection log via MS Teams. This integration of face-to-face sensor building (Periods 1–2) with online collaboration (Periods 3–4) exemplifies how blended learning leverages both in-person activities and digital platforms, enhancing flexibility, real-time data sharing, and collaborative problem-solving.

### 4.2 Pre–post survey: key gains

We administered 48 pre–post items (see Appendix A) focusing on scientific understanding, technology interest, collaboration, and problem-solving. Table 5 (short version) shows five representative items with statistically or practically significant improvements in math/science engagement and confidence.

**Table 5:** Selected pre–post changes (shortened version).

Survey item	Pre M	Post M	*p*-value	Cohen’s d
PP1_14 “I find math class content fun.”	2.80	3.20	0.028	0.57
PP1_16 “I enjoy reading math-related info/books.”	2.65	3.20	0.008	0.67
PP2_27 “I am confident I can solve math problems well.”	2.75	3.20	0.003	0.64
PP2_30 “I quickly grasp science content.”	2.65	3.20	0.001	0.80
PP4_46 “(Achievement level) Math”	3.25	3.85	0.019	0.65

See Appendix A for all items (PP1_x, PP2_x, PP3_x, PP4_x) with full descriptive statistics (means, SDs, *t*-values, *p*-values, effect sizes).

Students exhibited moderate-to-large gains in interest (PP1_14, PP1_16) and confidence (PP2_27, PP2_30). Notably, math achievement (PP4_46) also increased significantly, from 3.25 to 3.85 (*p* = 0.019, Cohen’s *d* = 0.65). Interview data confirmed that hands-on Arduino tasks made math/science more concrete and enjoyable. Several students remarked that collecting sensor data “gave math a real purpose” and “brought science to life,” aligning with these quantitative findings.

### 4.3 Satisfaction survey: highlights

Immediately after the program, students completed a 15-item satisfaction survey (renamed Q1 ~ Q15; see Appendix B). Table 6 highlights three key items: overall satisfaction, participation, and willingness to continue STEM.

**Table 6:** Selected post-program satisfaction results (shortened).

Item	Mean	SD
Q1 “Are you satisfied with the STEM class?”	4.3	0.66
Q3 “Did you actively participate in class activities?”	4.35	0.67
Q5 “Would you like to continue receiving STEM classes?”	3.9	0.91

Students rated the class highly (*M* = 4.3) and reported active involvement (*M* = 4.35). Although their desire for further STEM (*M* = 3.9) was slightly lower, the overall sentiment remained positive. This aligns with qualitative feedback indicating that practical sensor building and group tasks “made class more meaningful.”

### 4.4 Open-ended responses from structured interview questions

We also collected open-ended (OE) responses from three standardized questions administered during the semi-structured interviews. Although other open-ended discussions took place, these three questions (OE1, OE2, and OE3) were identical for all participants, allowing direct comparison of responses.

#### 4.4.1 OE1: differences from previous classes

Table 1 presents the frequency and percentage of each “difference” category mentioned by students. “Rank” indicates which aspect they perceived as the top difference (e.g., #1 difference).

About half of the respondents indicated “many group tasks” (OE1_1) as the biggest difference, while a “student-centered approach” (OE1_2) also appeared in 10% of responses. Students often remarked on collaboration and group dynamics, consistent with survey item related to teamwork (e.g., PP2_32).

#### 4.4.2 OE2: challenges

Table 2 summarizes the most frequently cited challenges in the STEM program. “Rank” indicates which challenge was noted as the most prominent (#1 challenge).

More than half the participants (55%) cited time constraints for hands-on tasks. Additionally, 35% encountered technical or coding difficulties. These challenges align with the reflection reports, where students described the trade-off between deep exploration and limited class hours.

#### 4.4.3 OE3: positive aspects

Table 3 shows how participants ranked each “positive aspect” from 1st (most important) through 6th (least important). The Mean Rank indicates overall priority: lower mean rank (closer to 1.0) implies higher perceived value.

Each column (1st–6th) shows how many students assigned that rank to the item. Mean Rank is calculated by multiplying each rank by its frequency, summing these products, and dividing by the total number of responses. Lower values (e.g., 2.60 for OE3_3) mean higher overall priority. For example, “Plenty of group activities” (OE3_3) was often rated top priority and thus yielded the lowest mean rank of 2.60. In contrast, “Opportunities to get info on science/tech careers” (OE3_6) scored 4.75, indicating it was less central for most students.

Group collaboration (OE3_3) and student-centered activities (OE3_2) emerged as the strongest positive themes, mirroring the OE1 findings on “many group tasks.” Students also appreciated the interdisciplinary nature (OE3_1) and how the class content related to real-life issues (OE3_5). Gathering dust data firsthand (e.g., coding Arduino sensors) enhanced problem-solving confidence, consistent with the pre-post survey gains in PP2 items (problem-solving self-efficacy).

### 4.5 Additional qualitative insights

A thematic analysis of interviews and reflection logs revealed key motivations and skill aspirations (Table 7). For instance:

**Table 7:** Thematic coding of selected interview excerpts.

Student (ID)	English translation	Themes	Interpretation
S4	By building a device using Arduino to measure fine particulate matter, I realized that everyday dust levels are higher than I thought. I created a poster and put it on my apartment board to inform others.	Environmental Awareness, Community Engagement	Recognizes seriousness of fine particulate matter in daily life; demonstrates initiative to share findings with neighbors, reflecting real-world application.
S5	I assumed coding only happens on a PC, but seeing how Arduino operated the dust kit was fascinating… Visualizing the data made me realize how serious the pollution really is.	Hands-on Coding, Problem Awareness	Gains practical coding exposure beyond the computer screen; direct sensor feedback heightened awareness of fine particulate matter.
S9	I took an Arduino class before, but working with a fine particulate matter sensor was new… It was satisfying to see it working, and I’d like to try other robot builds in the future.	Circuit Assembly, Future Exploration	Indicates intrinsic motivation following successful sensor assembly; desires further Arduino/robotics exploration.
S10	I was curious about the difference between my phone’s app and Naver Weather. Learning the sensor principles cleared that up… I realized coding is not as hard as I thought and is closely tied to life.	Practical Coding Insight, Sensor Principles	Student discovered how sensor methods can differ, found block-coding approachable, and recognized coding’s everyday potential.
S20	Fine particulate matter’s impact on the human body was worse than I thought, and actually building and measuring with the sensor made it feel real… I want to research this even further.	Health Awareness, Motivation for Further Study	Student shows heightened health consciousness and interest in deeper investigations, signaling potential for future STEM pursuits.

These findings suggest that hands-on design, authentic data measurement, and group synergy combined to foster problem-solving, spark environmental awareness, and strengthen collaboration.

## 5 Discussion

### 5.1 Alignment with previous research on STEM and blended learning

This study’s findings demonstrate that an Arduino-based STEM program can enhance disciplinary engagement, problem-solving, and collaborative skills, aligning with prior research suggesting that integrated STEM experiences promote both technical and non-technical competencies (English, 2016; Kelley and Knowles, 2016; National Research Council, 2012). In particular, the significant increases in math/science attitudes and confidence echo Bybee (2013), who underscores that authentic STEM challenges—such as building and deploying environmental sensors—foster deeper conceptual understanding. Moreover, students’ enthusiasm for collecting real-world data aligns with Tseng et al. (2013), whose project-based approach yielded similarly positive attitudes toward science, technology, engineering, and mathematics.

From a blended learning perspective, our results support the notion that hands-on, technology-facilitated tasks can boost content mastery and soft-skills (Bernard et al., 2014; Garrison and Vaughan, 2008). The program—combining face-to-face teamwork with digital tools (e.g., mBlock, Excel)—allowed students to move between autonomous exploration and group-based reflection, in line with Kintu et al. (2017), who emphasize how blended formats encourage self-regulated learning through diverse, flexible activities.

### 5.2 Soft skills and 21st-century competencies

This study, based on the 21st-century competency frameworks (Griffin and Care, 2015; Trilling and Fadel, 2009), utilized renamed survey item (e.g., PP2_32) and qualitative data (interviews and reflection logs) to uncover the processes by which students formulated hypotheses, debugged Arduino code, and discussed solutions. These activities align closely with critical thinking and collaboration, as emphasized by Trilling and Fadel (2009). The problem-solving rubric clarified iterative stages (defining issues, testing solutions, reflecting on outcomes), consistent with National Research Council (2012) guidelines on authentic scientific inquiry. Students’ initiatives—like measuring dust levels beyond the classroom—illustrate the broader societal dimension of problem-solving advocated by Griffin and Care (2015). By connecting technical tasks (e.g., sensor building) with real environmental concerns, the program cultivated applied thinking and civic responsibility.

## 6 Conclusion

### 6.1 Summary of key findings

The Arduino-based STEM program—focusing on “Measuring Fine particulate matter Using Arduino and Exploring Solutions”—demonstrated notable gains in student motivation, problem-solving abilities, and collaborative thinking. Hands-on sensor construction and iterative discussions of environmental issues allowed students to bridge theoretical knowledge with real-world applications, meeting the need for authentic STEM tasks (Bybee, 2013; National Research Council, 2012). Survey data showed positive shifts in attitudes and self-efficacy, while qualitative coding revealed deeper insight into how collaboration, communication, and civic awareness were strengthened through active engagement.

### 6.2 Limitations and future directions

Despite the promising outcomes, there are limitations to note.

(1) Single-Group Scope: Without a control group, attributing all improvements solely to the Arduino program is challenging (Kelley and Knowles, 2016). Future studies might include comparative or multi-site designs to validate broader applicability.
(2) Short Time Frame: The four-session format may not capture long-term skill retention or advanced coding proficiency (English, 2016). Extending the program could bolster self-regulation and more complex problem-solving strategies (Kintu et al., 2017).
(3) Measurement Tools: While certain survey items were mapped to collaboration and problem-solving, a more targeted assessment (beyond self-report) could provide finer-grained data on soft skill development.

Future research might investigate longitudinal effects on career pathways, environmental stewardship, or coding autonomy. Examining diverse student demographics could also provide valuable insights into replicating or scaling this approach.

## Data Availability

The original contributions presented in the study are included in the article/Supplementary material, further inquiries can be directed to the corresponding author.
