# Supplementary material for: Arduino-based fine particulate matter STEM program: enhancing problem-solving and collaboration in a post-pandemic blended high school setting
Source: Front Psychol. 2025 Apr 22;16:1524777. doi: 10.3389/fpsyg.2025.1524777 (PMC12053152; doi:10.3389/fpsyg.2025.1524777)
Supplement: Supplementary file 1 [file Supplementary_file_1.docx]

# Appendix A Full Pre–Post Survey Results

**Table A1.** Complete Pre–Post Survey Items with Descriptive Statistics and Effect Sizes

| Item Code | Survey Question | Pre-  Mean | Pre-  SD | Post-  Mean | Post-  SD | Mean Diff. | t-  value | p-value | Cohen’s d |
| --- | --- | --- | --- | --- | --- | --- | --- | --- | --- |
| PP1_1 | I find the content of science class interesting. | 3.15 | 0.59 | 3.15 | 0.75 | 0 | 0.00 | 1.0000 | 0.00 |
| PP1_2 | I am confident in my ability to understand math content. | 3.1 | 0.55 | 3.35 | 0.59 | 0.25 | -2.03 | 0.0563 | 0.44 |
| PP1_3 | I find math-related activities (math experience activities, solving math quizzes, or reading math-related materials) fun. | 2.95 | 0.69 | 3.1 | 0.79 | 0.15 | -0.68 | 0.5054 | 0.20 |
| PP1_4 | Having a career related to science is a great thing. | 3.45 | 0.51 | 3.6 | 0.60 | 0.15 | -0.90 | 0.3793 | 0.27 |
| PP1_5 | I enjoy studying science. | 3.1 | 0.64 | 3.1 | 0.79 | 0 | 0.00 | 1.0000 | 0.00 |
| PP1_6 | I am confident in my ability to understand science content. | 3.05 | 0.51 | 3 | 0.79 | -0.05 | 0.33 | 0.7481 | -0.07 |
| PP1_7 | I enjoy looking up and reading science-related information or books. | 2.9 | 0.85 | 2.9 | 0.91 | 0 | 0.00 | 1.0000 | 0.00 |
| PP1_8 | I like science. | 3.3 | 0.47 | 3.3 | 0.57 | 0 | 0.00 | 1.0000 | 0.00 |
| PP1_9 | I am interested in a career related to mathematics. | 2.75 | 0.64 | 3.05 | 0.69 | 0.3 | -2.04 | 0.0553 | 0.45 |
| PP1_10 | I find science-related activities (visiting science museums, participating in science events, reading science materials) fun. | 3.1 | 0.55 | 3.15 | 0.67 | 0.05 | -0.44 | 0.6663 | 0.08 |
| PP1_11 | I share and use science lab equipment amicably with other classmates. | 3.55 | 0.51 | 3.5 | 0.51 | -0.05 | 0.57 | 0.5770 | -0.10 |
| PP1_12 | It is important to exchange opinions with classmates when doing group work in science class. | 3.4 | 0.60 | 3.5 | 0.61 | 0.1 | -0.52 | 0.6058 | 0.17 |
| PP1_13 | Studying science helps with learning in high school or college. | 3.4 | 0.50 | 3.4 | 0.60 | 0 | 0.00 | 1.0000 | 0.00 |
| PP1_14 | I find the content of math class interesting. | 2.8 | 0.70 | 3.2 | 0.70 | 0.4 | -2.37 | 0.0284 | 0.57 |
| PP1_15 | I listen attentively to my classmate’s presentation in math class. | 3.25 | 0.44 | 3.45 | 0.51 | 0.2 | -1.71 | 0.1036 | 0.42 |
| PP1_16 | I enjoy looking up and reading math-related information or books. | 2.65 | 0.81 | 3.2 | 0.83 | 0.55 | -2.98 | 0.0077 | 0.67 |
| PP1_17 | Math is helpful for studying other subjects. | 3.2 | 0.52 | 3.25 | 0.64 | 0.05 | -0.33 | 0.7481 | 0.09 |
| PP1_18 | In math class, I respect the opinions of classmates who differ from mine. | 3.45 | 0.51 | 3.45 | 0.51 | 0 | 0.00 | 1.0000 | 0.00 |
| PP1_19 | Having a career related to mathematics is a great thing. | 3.2 | 0.62 | 3.4 | 0.60 | 0.2 | -1.00 | 0.3299 | 0.33 |
| PP1_20 | In science class, when I express an opinion, I also consider other classmates’ perspectives. | 3.35 | 0.49 | 3.45 | 0.51 | 0.1 | -0.70 | 0.4936 | 0.20 |
| PP2_21 | I enjoy studying math. | 2.95 | 0.60 | 3.1 | 0.72 | 0.15 | -1.00 | 0.3299 | 0.23 |
| PP2_22 | I actively exchange opinions with my classmates during math class. | 2.9 | 0.85 | 3.2 | 0.70 | 0.3 | -1.55 | 0.1372 | 0.39 |
| PP2_23 | I actively exchange opinions with my classmates during science class. | 2.9 | 0.72 | 3.1 | 0.79 | 0.2 | -1.45 | 0.1625 | 0.27 |
| PP2_24 | I think science is easy. | 2.2 | 0.52 | 2.55 | 0.94 | 0.35 | -1.51 | 0.1488 | 0.46 |
| PP2_25 | I actively express my thoughts in math class. | 2.8 | 0.77 | 2.85 | 0.93 | 0.05 | -0.27 | 0.7894 | 0.06 |
| PP2_26 | I listen attentively to my classmate’s presentation in science class. | 3.35 | 0.49 | 3.45 | 0.51 | 0.1 | -1.00 | 0.3299 | 0.20 |
| PP2_27 | I am confident that I can solve math problems well. | 2.75 | 0.72 | 3.2 | 0.70 | 0.45 | -3.33 | 0.0035 | 0.64 |
| PP2_28 | I quickly grasp math content. | 2.85 | 0.67 | 3.15 | 0.67 | 0.3 | -2.35 | 0.0298 | 0.45 |
| PP2_29 | Science is helpful for studying other subjects. | 3.15 | 0.59 | 3.35 | 0.49 | 0.2 | -1.71 | 0.1036 | 0.37 |
| PP2_30 | I quickly grasp science content. | 2.65 | 0.67 | 3.2 | 0.70 | 0.55 | -4.07 | 0.0007 | 0.80 |
| PP2_31 | Scientific knowledge is useful in daily life. | 3.3 | 0.47 | 3.45 | 0.51 | 0.15 | -1.37 | 0.1864 | 0.31 |
| PP2_32 | It is important to exchange opinions with classmates when doing group work in math class. | 3.2 | 0.62 | 3.5 | 0.51 | 0.3 | -2.35 | 0.0298 | 0.53 |
| PP2_33 | Studying math helps with learning in high school or college. | 3.5 | 0.51 | 3.45 | 0.60 | -0.05 | 0.29 | 0.7715 | -0.09 |
| PP2_34 | I like mathematics. | 2.8 | 0.62 | 3.15 | 0.75 | 0.35 | -1.93 | 0.0692 | 0.51 |
| PP2_35 | I am confident that I can solve science problems well. | 2.75 | 0.79 | 3 | 0.86 | 0.25 | -1.56 | 0.1351 | 0.30 |
| PP2_36 | I am interested in careers related to science. | 3.15 | 0.49 | 3.4 | 0.50 | 0.25 | -2.03 | 0.0563 | 0.50 |
| PP2_37 | I actively express my thoughts in science class. | 2.75 | 0.79 | 3.1 | 0.91 | 0.35 | -1.58 | 0.1297 | 0.41 |
| PP2_38 | I think math is easy. | 2.35 | 0.75 | 2.75 | 0.91 | 0.4 | -1.80 | 0.0880 | 0.48 |
| PP2_39 | When I state an opinion in math class, I also consider my classmates’ perspectives. | 3.2 | 0.52 | 3.45 | 0.51 | 0.25 | -1.75 | 0.0961 | 0.48 |
| PP2_40 | Mathematical knowledge is useful in daily life. | 3 | 0.65 | 3.3 | 0.73 | 0.3 | -1.67 | 0.1105 | 0.43 |
| PP3_41 | (Degree of liking) Korean | 3.6 | 0.82 | 3.5 | 0.95 | -0.1 | 0.49 | 0.6295 | -0.11 |
| PP3_42 | (Degree of liking) Math | 3.95 | 0.76 | 3.9 | 0.79 | -0.05 | 0.44 | 0.6663 | -0.06 |
| PP3_43 | (Degree of liking) Science | 4 | 0.73 | 4 | 0.79 | 0 | 0.00 | 1.0000 | 0.00 |
| PP3_44 | (Degree of liking) Technology | 3.45 | 0.89 | 3.3 | 1.22 | -0.15 | 0.51 | 0.6142 | -0.14 |
| PP4_45 | (Achievement level) Korean | 3.25 | 0.91 | 3.5 | 1.00 | 0.25 | -1.42 | 0.1713 | 0.26 |
| PP4_46 | (Achievement level) Math | 3.25 | 0.85 | 3.85 | 0.99 | 0.6 | -2.56 | 0.0190 | 0.65 |
| PP4_47 | (Achievement level) Science | 3.15 | 0.88 | 3.5 | 1.10 | 0.35 | -2.33 | 0.0308 | 0.35 |
| PP4_48 | (Achievement level) Technology | 3.25 | 0.97 | 3.2 | 1.20 | -0.05 | 0.20 | 0.8409 | -0.05 |

# Appendix B Full Post-Program Satisfaction Survey Items

**Table B1.** Post-Program Satisfaction Survey Results

| Item Code | Survey Question | Mean | SD |
| --- | --- | --- | --- |
| Q1 | Are you satisfied with the STEM class? | 4.30 | 0.66 |
| Q2 | Was the STEM class fun? | 4.20 | 0.70 |
| Q3 | Did you actively participate in STEM class activities? | 4.35 | 0.67 |
| Q4 | What do you think about the level/content of the STEM class? | 3.65 | 0.88 |
| Q5 | Would you like to continue receiving STEM classes in the future? | 3.90 | 0.91 |
| Q6 | I have come to find science class more interesting. | 3.85 | 0.93 |
| Q7 | I tried to apply knowledge from various subjects simultaneously when solving problems. | 4.00 | 0.92 |
| Q8 | I participated actively and enthusiastically in class. | 4.20 | 0.89 |
| Q9 | I had logical discussions with my classmates. | 4.10 | 0.85 |
| Q10 | I expressed my ideas to other classmates. | 4.35 | 0.81 |
| Q11 | I listened to and respected other classmates’ opinions. | 4.55 | 0.60 |
| Q12 | I have come to realize the importance of collaborating with my classmates. | 4.50 | 0.61 |
| Q13 | I have become more considerate of my classmates. | 4.45 | 0.60 |
| Q14 | I am no longer afraid of failing, and I feel more motivated to take on challenges. | 3.85 | 0.99 |
| Q15 | I have become interested in careers related to science and technology. | 3.95 | 0.89 |
